# Appearances Can Be Deceptive: Revealing a Hidden Viral Infection with Deep Sequencing in a Plant Quarantine Context

**DOI:** 10.1371/journal.pone.0102945

**Published:** 2014-07-25

**Authors:** Thierry Candresse, Denis Filloux, Brejnev Muhire, Charlotte Julian, Serge Galzi, Guillaume Fort, Pauline Bernardo, Jean-Heindrich Daugrois, Emmanuel Fernandez, Darren P. Martin, Arvind Varsani, Philippe Roumagnac

**Affiliations:** 1 INRA, UMR 1332 Biologie du Fruit et Pathologie, CS 20032, 33882 Villenave d'Ornon Cedex, France; 2 Université de Bordeaux, UMR 1332 Biologie du Fruit et Pathologie, CS 20032, 33882 Villenave d'Ornon Cedex, France; 3 CIRAD, UMR BGPI, Campus International de Montferrier-Baillarguet, 34398 Montpellier Cedex-5, France; 4 Computational Biology Group, Institute of Infectious Diseases and Molecular Medicine, University of Cape Town, Cape Town, South Africa; 5 School of Biological Sciences and Biomolecular Interaction Centre, University of Canterbury, Christchurch, New Zealand; 6 Department of Plant Pathology and Emerging Pathogens Institute, University of Florida, Gainesville, Florida, United States of America; 7 Electron Microscope Unit, Division of Medical Biochemistry, Department of Clinical Laboratory Sciences, University of Cape Town, Observatory, South Africa; Washington State University, United States of America

## Abstract

Comprehensive inventories of plant viral diversity are essential for effective quarantine and sanitation efforts. The safety of regulated plant material exchanges presently relies heavily on techniques such as PCR or nucleic acid hybridisation, which are only suited to the detection and characterisation of specific, well characterised pathogens. Here, we demonstrate the utility of sequence-independent next generation sequencing (NGS) of both virus-derived small interfering RNAs (siRNAs) and virion-associated nucleic acids (VANA) for the detailed identification and characterisation of viruses infecting two quarantined sugarcane plants. Both plants originated from Egypt and were known to be infected with Sugarcane streak Egypt Virus (SSEV; Genus *Mastrevirus*, Family *Geminiviridae*), but were revealed by the NGS approaches to also be infected by a second highly divergent mastrevirus, here named Sugarcane white streak Virus (SWSV). This novel virus had escaped detection by all routine quarantine detection assays and was found to also be present in sugarcane plants originating from Sudan. Complete SWSV genomes were cloned and sequenced from six plants and all were found to share >91% genome-wide identity. With the exception of two SWSV variants, which potentially express unusually large RepA proteins, the SWSV isolates display genome characteristics very typical to those of all other previously described mastreviruses. An analysis of virus-derived siRNAs for SWSV and SSEV showed them to be strongly influenced by secondary structures within both genomic single stranded DNA and mRNA transcripts. In addition, the distribution of siRNA size frequencies indicates that these mastreviruses are likely subject to both transcriptional and post-transcriptional gene silencing. Our study stresses the potential advantages of NGS-based virus metagenomic screening in a plant quarantine setting and indicates that such techniques could dramatically reduce the numbers of non-intercepted virus pathogens passing through plant quarantine stations.

## Introduction

When attempting to prevent the spread of plant diseases, comprehensive inventories of viral diversity are fundamental both for effective quarantine and sanitation efforts, and to ensure that plant materials within biological resource centres (BRCs) can be safely distributed [1], [2]. Detection of pathogens is one of the most critical quarantine and BRC operations. Ideally, the tools used for this purpose must be both sensitive enough to accurately detect the presence of even extremely low amounts of pathogen nucleic acids or proteins, and provide sufficient specific information to identify the genetic variants/strains of whatever pathogens are present.

The major challenge of using classical nucleic acid sequence-informed detection tools such as polymerase chain reaction (PCR) or Southern hybridisation assays, is that despite being highly sensitive, these techniques are generally either species or, at best, genus-specific. In addition, such tools lack the capacity to detect, let alone identify, pathogens that are unknown, poorly characterized or highly variable. Although it might be argued that the most economically important pathogens tend to be well characterized and that it is therefore not a serious issue that many of the more obscure pathogens go undetected, it is becoming better appreciated that the “importance” of any particular pathogenic microbe is very difficult to define. Specifically, the environmental and economic impacts of a particular pathogen can vary widely with varying climatic and ecological conditions and there are large numbers of microbes that are presently not classified as pathogens (or at least which are not noticeably pathogenic to humans or to domesticated plants and animals), which will eventually emerge as important future pathogens [3]. Also, since non-domesticated plant and animal species and countless numbers of microbes which contribute to natural terrestrial ecosystems [4]–[6] can also potentially be threatened by exotic pathogens, the unconstrained global dissemination of apparently harmless fungi, viruses and bacteria could have serious environmental and economic impacts.

Whereas “sequence-dependent” microbial detection methods, which are generally based on PCR or nucleic acid hybridisation, can only be used to target known pathogens, sequence-independent next generation sequencing (NGS) based approaches can potentially provide an ideal platform for identifying almost all known and unknown microbes present in any particular host organism [5], [7]–[9]. Coupled with innovative sample processing procedures, “metagenomics” applications of NGS [10] have already enabled the identification of novel pathogens through the rapid and comprehensive characterization of microbial strains and isolates within environmental and host tissue samples [9], [11].

In addition to numerous applications in the study of animal infecting viruses, NGS-based metagenomics approaches have also been used to detect plant infecting viruses [12]. Three main classes of nucleic-acids have been targeted by such analyses: (1) virion-associated nucleic acids (VANA) purified from viral particles [13], [14]; (2) double-stranded RNAs (dsRNA) [15]; and (3) virus-derived small interfering RNAs (siRNAs) [16]. Large numbers of both known and new plant and fungus infecting DNA and RNA viruses and viroids have been detected using these approaches [12], [17]–[21].

A major shortcoming of these metagenomic approaches is, however, that they remain technically cumbersome and too expensive for routine diagnostic applications on collections of eukaryotic hosts - even if barcoded primers are used to bulk-sequence pooled samples from multiple sources [15]. Although prohibitive for high throughput diagnostics, the costs of NGS in the context of viral diversity research are often offset by the vast volumes of useful data that can be generated on viral population dynamics, co-infections, mutation frequencies and genetic recombination [22]–[24].

Here we describe the application of siRNA- and VANA-targeted NGS approaches to the analyses of two Egyptian sugarcane plants maintained for a number of years at the CIRAD Sugarcane Quarantine Station in Montpellier, France. These plants were both known to be infected with *Sugarcane streak Egypt Virus* (SSEV; Family *Geminiviridae*, Genus *Mastrevirus*) and were maintained for use as positive controls during the application of diagnostic tools for SSEV detection in sugarcane plants passing through the quarantine station. Using the siRNA- and VANA-targeted NGS approaches, we discovered and characterized a novel highly divergent mastrevirus from these two plants. This novel virus was also identified in other sugarcane plants originating from Sudan that exhibited white spots on the base of their leaf blades that become fused laterally, so as to appear as chlorotic stripes. Accordingly, we have proposed naming this virus Sugarcane white streak Virus (SWSV). In addition, we present a detailed analysis of siRNAs derived from the SWSV and SSEV variants infecting the two analysed sugarcane plants.

## Materials and Methods

### Plant material and sugarcane quarantine DNAs collection

Leaves presenting typical symptoms of sugarcane streak disease were sampled from two sugarcane plants that had previously been found to be infected with *Sugarcane streak Egypt virus* (SSEV) and had been kept in a quarantine greenhouse at the CIRAD Sugarcane Quarantine Station, in Montpellier, France. The two sugarcane plants, VARX and USDA (which was initially maintained at USDA-APHIS Plant Germplasm Quarantine before being transferred to CIRAD in 2007), were both initially collected in Egypt during two independent sampling surveys in the late 1990s [25], [26]. These sampling surveys were both carried out on experimental stations and commercial lands in close collaboration with Egyptian authorities (Sugar Crop Research Institute (SCRI), Dr Abdel Wahab I. Allam (Director of SCRI) regarding VARX; and Agricultural Genetic Engineering Research Institute, Dr N.A. Abdallah, and Dr M.A. Madkour regarding USDA). In addition, leaves from six sugarcane plants originating from Sudan (B0065, B0067, B0069, D0002, D0003 and D0005, Table S1) and maintained at the CIRAD Sugarcane Quarantine Station were also used (Material Transfer Agreements between CIRAD and Kenana Sugar Co. Ltd). DNAs from these six plants were extracted using the DNeasy Plant Mini Kit (Qiagen). In addition, DNA was extracted from an additional 18 frozen leaf samples (−20°C), including 17 samples originating from Sudanese sugarcane plants, which had passed through the Montpellier Quarantine station between 2000 and 2009 and one which had been obtained from a sugarcane seedling grown from sugarcane true seeds [fuzz] developed in Guadeloupe from a biparental cross involving plants H70-6957 and B86-049 using the DNeasy Plant Mini Kit (Table S1).

### VANA extraction from viral particles, cDNA amplification and sequencing

One gram of leaf material from the VARX and USDA plants were ground in Hanks' buffered salt solution (HBSS) (1∶10) with four ceramic beads (MP Biomedicals, USA) using a tissue homogeniser (MP biomedicals, USA). The homogenised plant extracts were centrifuged at 3,200×g for 5 min and 6 ml of the supernatants were further centrifuged at 8,228×g for 3 min. The resulting supernatants were then filtered through a 0.45 µm sterile syringe filter. The filtrate was then centrifuged at 148,000×g for 2.5 hrs at 4°C to concentrate viral particles. The resulting pellet was resuspended overnight at 4°C in 200 µl of HBSS. Non-encapsidated nucleic acids were eliminated by adding 15 U of bovine pancreas DNase I (Euromedex) and 1.9 U of bovine pancreas RNase A (Euromedex, France) followed by incubation at 37°C for 90 min. Total nucleic acids were finally extracted from virions using a NucleoSpin 96 Virus Core Kit (Macherey-Nagel, Germany) following the manufacturer's protocol. The amplification of extracted nucleic acids was performed as described by Victoria *et al*. [14] and aimed at detecting both RNA and DNA viruses. Reverse transcriptase priming and amplification of nucleic acids were used for detecting RNA viruses. A Klenow Fragment step was included in the protocol in order to detect DNA viruses as demonstrated by Froussard [27]. Briefly, viral cDNA synthesis was performed by incubation of 10 µl of extracted viral nucleic acids with 100 pmol of primer DoDec (5′-CCT TCG GAT CCT CCN NNN NNN NNN NN-3′) at 85°C for 2 min. The mixture was immediately placed on ice. Subsequently, 10 mM dithiothreitol, 1 mM of each deoxynucloside triphosphate (dNTP), 4 µl of 5× Superscript buffer, and 5 U of SuperScript III (Invitrogen, USA) were added to the mixture (final volume of 20 µl), which was then incubated at 25°C for 10 min, followed by 42°C incubation for 60 min and 70°C incubation for 5 min before being placed on ice for 2 min. cDNAs were purified using the QiaQuick PCR cleanup kit (Qiagen). Priming and extension was then performed using Large (Klenow) Fragment DNA polymerase (Promega). First, 20 µl of cDNA in the presence of 4.8 µM of primer DoDec were heated to 95°C for 2 min and then cooled to 4°C. 2.5 U of Klenow Fragment, 10X Klenow reaction buffer and 0.4 mM of each dNTP (final volume of 25 µl) were added. The mixture was incubated at 37°C for 60 min followed by 75°C enzyme heat inactivation for 10 min. PCR amplification was carried out using 5 µl of the reaction described above in a 20 µl reaction containing 2 µM primer (LinkerMid50 primer for VARX: 5′-ATC GTA GCA GCC TTC GGA TCC TCC-3′ and LinkerMid52 primer for USDA: 5′-ATG TGT CTA GCC TTC GGA TCC TCC-3′), and 10 µl of HotStarTaq Plus Master Mix Kit (Qiagen). The following cycling conditions were used: one cycle of 95°C for 5 min, five cycles of 95°C for 1 min, 50°C for 1 min, 72°C for 1.5 min, 35 cycles of 95°C for 30 sec, 50°C for 30 sec, 72°C for 1.5 min +2 sec at each cycle. An additional final extension for 10 min at 72°C was then performed. DNA products were pooled (VARX and USDA products and 94 additional products obtained from other quarantine samples), cleaned up using the Wizard SV Gel and PCR Clean-Up System (Promega) and sequenced on 1/8^th^ of a 454 pyrosequencing plate using GS FLX Titanium reagents (Beckman Coulter Cogenics, USA).

### siRNA extraction and sequencing

The nucleic acid extraction and sequencing approach of Kreuze *et al*. [16] was used with slight modifications. Total RNAs were extracted from 100mg of VARX fresh leaf material using Trizol (Invitrogen) following the manufacturer's instructions. Small RNA libraries were directly generated from total RNAs. Small RNAs ligated with 3′ and 5′ adapters were reverse transcribed and PCR amplified (30 sec at 98°C; [10 sec at 98°C, 30 sec at 60°C, 15 sec at 72°C] ×13 cycles; 10 min at 72°C) to create cDNA libraries selectively enriched in fragments having adapter molecules at both ends. The last step was an acrylamide gel purification of the 140–150 nt amplified cDNA constructs (corresponding to cDNA inserts from siRNAs +120 nt from the adapters). Small RNA libraries were checked for quality and quantified using a 2100 Bioanalyzer (Agilent). The library was then sequenced on one lane of a HiSeq Illumina as single-end 50 base reads.

### Sequence assembly

Analyses of reads produced by either Illumina (siRNA sequencing) or 454 GS FLX Titanium (Amplified-VANA sequencing) were performed using CLC Genomics Workbench 5.15. *De novo* assemblies of contigs were performed with a minimal contig size set at 100 bp and 200 bp for Illumina and 454 GS FLX Titanium reads, respectively. *A posteriori* mapping of reads against the complete genomes of SWSV (once the full genome had been cloned and sequenced) or SSEV or against parts of these genomes were also performed using CLC Genomics Workbench 5.15. Primary sequence outputs have been deposited in the sequence read archive of GenBank (accession numbers: VANA_USDA dataset: SRR1207274; VANA_VARX dataset: SRR1207275; siRNA_VARX dataset: SRR1207277).

### SWSV genome amplification, cloning and sequencing

Two partially overlapping SWSV specific PCR primer pairs were designed so as to avoid any potential cross-hybridization to 63 representative species of the family *Geminiviridae,* including SSEV. These two primer pairs (pair1: SWSV_F1 forward primer 5′-GCT GAA ACC TAT GGC AAA GA-3′ and SWSV-R1 reverse primer 5′-AGC CTC TCT ACA TCC TTT GC-3′; and pair2 ECORI-1F forward primer 5′-GAA TTC CCA GAG CGT GGT A-3′ and ECORI-2R reverse primer 5′-GAG TTG AAT TCC GGT ACC AAG GAC-3′) were complementary to sequences within the *rep* gene of SWSV. Total DNAs from the two sugarcane plants described above (VARX and USDA) were extracted using the DNeasy Plant Mini Kit (Qiagen) and screened for SWSV using the two pairs of primers and GoTaq Hot Start Master Mix (Promega) following the manufacturer's protocol. Amplification conditions consisted of an initial denaturation at 95°C for 2 min, 35 cycles at 94°C for 10 sec, 55°C for 30 sec, 68°C for 3 min, and a final extension step at 68°C for 10 min. Amplification products of ∼2.8 Kbp were gel purified, ligated to pGEM-T (Promega) and sequenced by standard Sanger sequencing using a primer walking approach.

Reverse transcriptase priming and amplification of nucleic acids were carried out in order to detect the intron of the *rep* gene. Total RNAs from VARX were extracted using the RNeasy Plant Mini Kit (Qiagen). DNase treatment of extracted RNAs was carried out using RQ1 RNase-Free DNase (Promega) following the manufacturer's protocol. Viral cDNA synthesis was performed by incubation of 1 µl of DNase treated RNAs with 15 µl of RNase free water, 0.6 µM of each primers (SWSV_F2: 5′-ACC ATG TGC TGC CAG TAA TT-3′ and ECORI-2R: 5′-GAG TTG AAT TCC GGT ACC AAG GAC-3′), and 0.4 mM of mixed deoxynucloside triphosphate (dNTPs), 5 µl of 5X Qiagen OneStep RT-PCR Buffer and 1 µl of Qiagen OneStep RT-PCR Enzyme Mix. Tubes were first placed at 50°C for 30 min for cDNA synthesis. PCR amplification was then carried out using the following cycling conditions: One cycle of 95°C for 15 min, 35 cycles of 94°C for 1 min, 55°C for 1 min, 72°C for 1 min. An additional final extension for 10 min at 72°C was then performed. Amplification products were gel purified, ligated to pGEM-T (Promega) and sequenced by standard Sanger sequencing.

### PCR detection tests

DNAs extracted from 17 sugarcane plants originating from Sudan kept at −20°C or six freshly extracted from plants maintained at the CIRAD Sugarcane Quarantine Station were screened for SWSV. DNA extracted from one sugarcane seedling grown from true seeds (fuzz) was also screened for SWSV. PCR amplification was carried out using the two pairs of primers described above (SWSV_F1 and SWSV_R1; ECORI-1F and ECORI-2R) using GoTaq Hot Start Master Mix (Promega) following the manufacturer's protocol. Amplification products of ∼2.8 Kbp were gel purified, ligated to pGEM-T (Promega) and sequenced as described above. Plants infected with SWSV were also screened for all known sugarcane-infecting mastreviruses:*Sugarcane streak Egypt Virus*, *Sugarcane streak virus*, *Maize streak virus*, *Sugarcane streak Reunion virus*, *Eragrostis streak virus* and *Saccharum streak virus*. PCR amplification was carried out using 1 µl of DNA template in a 25 µl reaction containing 0.2 µM of each broad spectrum primer (SSV_1732F: 5′-CAR TCV ACR TTR TTY TGC CAG TA-3′ and SSV_2176R: 5′-GAR TAC CTY TCH ATG MTH CAG A-3′) and GoTaq Hot Start Master Mix (Promega) following the manufacturer's protocol. The following cycling conditions were used: One cycle of 95°C for 2 min, 35 cycles of 94°C for 1 min, 53°C for 1 min, 72°C for 1 min. An additional final extension for 10 min at 72°C was then performed.

### Sequence analyses

Six complete genomes of the novel mastrevirus were recovered from plants VARX, USDA, A0037, B0069, D0005 and E0144 (Table S1) and were aligned with the genomes of representative mastreviruses using MUSCLE (with default settings) [28]. Similarly, the predicted replication associated protein (Rep) and capsid protein (CP) amino acid sequences encoded by the viruses within the full-genome dataset were also aligned using MUSCLE. Maximum likelihood phylogenetic trees were inferred for the full genomes (TN93+G+I nucleotide substitution model chosen as the best-fit using jModelTest [29]), Rep (WAG+G+F amino acid substitution model chosen as the best-fit using ProtTest [30]) and CP (rtREV+G+F amino acid substitution model chosen as the best-fit using ProtTest) datasets with PHYML [31]. Approximate likelihood ratio tests (aLRT) were used to infer relative supports for branches (with branches having <80% support being collapsed). All pairwise identity analysis of the full genome nucleotide sequences, capsid protein (CP) amino acid sequences, replication associated protein (Rep) amino acid sequences and movement protein (MP) amino acid sequences were carried out using the MUSCLE-based pairwise alignment and identity calculation approach implemented in SDT v1.0 [32]. The full genome sequence alignment of representative mastrevirus genome sequences together with SWSV was used to detect evidence of recombination in SWSV using RDP 4.24 with default settings [33]. Sequences are deposited in GenBank under accession numbers (SWSV-A [SD-VARX-2013] - KJ187746; SWSV-A [SD -USDA-2013] - KJ187745; SWSV-B [SD -B0069-2013] – KJ210622; SWSV-B [SD -D0005-2013] - KJ187747; SWSV-B [SD -E0144-2013] - KJ187748 and SWSV-C [SD -A0037-2013] - KJ187749).

### Test for associations between siRNAs and SWSV/SSEV genomic and transcript secondary structures

The SWSV/SSEV full genome sequences and predicted unspliced complementary and virion strand transcripts were separately folded using Nucleic Acid Secondary Structure Predictor [34], with the sequence conformation set as circular DNA, at a temperature of 25°C. NASP generates a list of all secondary structures detectable within given DNA or RNA sequences and through simulations it demarcates a set of structures referred to as a “high confidence structure set” (HCSS), that confers a higher degree of thermodynamic stability (lower free energy) to the sequences than what would be expected to be achievable by randomly generated sequences with the same base composition (with a p< = 0.05).

Given the genomic coordinates of pairing nucleotides within the HCSS, we investigated whether there was any significant trend for more reads (looking both at all reads collectively and at the 21 nt, 22 nt, 23 nt and 24 nt long reads separately) occurring within secondary structures predicted to occur within (i) the full genomes, (ii) the virion-strand transcripts and (iii) the complementary-strand transcripts. The reads were mapped to the secondary structures and we counted how many nucleotides were located at paired and unpaired sites. While Kolmogorov-Smirnov tests (implemented in R; www.r-project.org) were used to determine whether the distribution of reads between paired and unpaired sites were different, Wilcoxon rank-sum tests (also implemented in R, www.r-project.org) indicated whether there were significantly more reads at paired sites compared to unpaired sites and *vice versa*. Whereas the Kolmogorov-Smirnov tests were used to indicate whether any associations existed between siRNA locations and base pairing within nucleic acid secondary structures, the Wilcoxon rank-sum tests were used to determine whether detected associations were positive (siRNAs tended to occur at structured sites) or negative (siRNAs tended to occur outside of structured sites).

## Results

### 454-based sequencing of VANA from the VARX and USDA sugarcane samples

This approach was used in an attempt to detect both RNA and DNA viruses that may be present in the two sugarcane plants [27]. A total of 2612 and 1635 reads were respectively obtained from the VARX and USDA plants following length and quality filtering. One hundred and eight and 18 contigs were produced by *de novo* assembly from the VARX- and USDA-derived reads, respectively. Two contigs from the VARX plant (2706 nt and 412 nt) and two from the USDA plant (2706 nt and 649 nt), encoded proteins with between 91 and 100% sequence identity with previously described SSEV proteins (Table1). BLASTx analysis revealed that an additional two contigs from the VARX plant (2122 and 127 nt) and three contigs from the USDA plant (1836, 196 and 312 nt) were homologous with known mastreviruses but were nevertheless only distantly related to mastrevirus sequences currently deposited in GenBank (Table1).

**Table 1:** Lengths, numbers of reads and BlastX analysis results for VANA 454 *de novo* contigs from sugarcane plants VARX and USDA with detectable homology to mastreviral sequences.

Sample	Contig	Contig length (bp)	Number of reads	BlastX Virus	BlastXLocus	BlastX e-value	Percent identity
VARX	#1	2706	1387	SSEV (NP_045945)	RepA	0.00	100%
	#2	2122	470	DDSMV (YP_003915158)	CP	3.84E–56	70%
	#3	412	11	SSEV (AAC98076)	MP	2.07E–8	95,2%
	#7	127	1	BCSMV (YP_004089628)	RepA	1.72e–10	71%
USDA	#1	2706	1128	SSEV (NP_04945)	RepA	9.20E–177	99.2%
	#2	1836	82	DDSMV (YP_003915158)	CP	1.04E–56	48.8%
	#3	649	12	SSEV (NP_04945)	RepA	2.80E–66	91.1%
	#4	196	37	MSV (CAA10092)	RepA	1.34E–8	56.2%
	#13	312	83	SSEV (AAF76868)	RepA	1.65E–30	84.3%

Acronyms used are as follows: SSEV (Sugarcane streak Egypt virus), DDSMV (Digitaria didactyla striate mosaic virus), BCSMV (Bromus catharticus striate mosaic virus), MSV (Maize streak virus).

**Figure 1:**
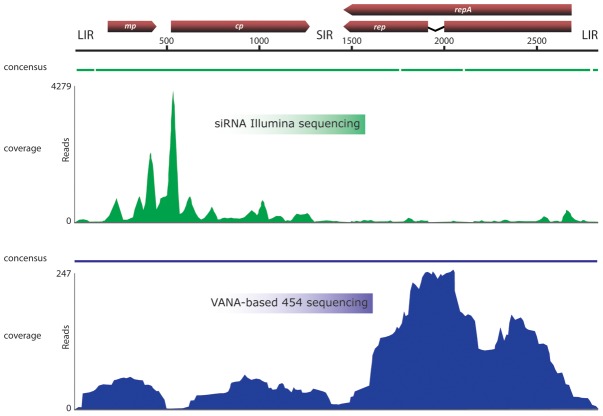
SWSV genome coverage following NGS. The genomic organization of SWSV is schematically shown above the graph. While relative degrees of coverage achieved after *a posteriori* mapping of reads produced by Illumina-based siRNA sequencing against the SWSV genome is indicated in green, the coverage achieved after mapping reads produced by 454 GS FLX Titanium-based VANA sequencing is indicated in blue.

*A posteriori* mapping of VANA 454 reads obtained from the VARX and USDA plants against the complete SWSV genome (see below), revealed that 23.9% (625/2612) and 16.1% (264/1635) of the total reads were derived from this genome and that these yielded complete genome coverage at an average depth of 81X and 29X, respectively. Interestingly, a ∼120 nt long region of very low coverage (<4X) was identified, which mapped to the large intergenic region (LIR) of the SWSV genome (Figure 1).

A mapping analysis performed with the genome of SSEV indicated that the corresponding values were 53.5% of reads (1398/2612, 159X average coverage depth) and 75% of reads (1227/1635, 138X coverage) for the VARX and USDA plants, respectively (Figure S1).

### siRNA Illumina sequencing from the VARX sugarcane plant

A total of 15,275,640 raw reads were generated from the VARX sugarcane sample, which were then filtered down to 3,945,108 high quality reads in the 21 to 24 nt size range of siRNAs. From these reads, 226 contigs were obtained by *de novo* assembly, six of which showed significant degrees of similarity to mastreviruses based on BLASTx [35] searches (Table2). Of these six contigs, two (contigs #121 and #176) had a high degree of identity to SSEV while the remaining four were more distantly related to known mastreviruses. Three of these four contigs (contigs #44, #86 and #101) apparently corresponded with a mastrevirus capsid protein (CP) gene and the other one (contigs #79) with a movement protein (MP) gene, while the cumulative contig length of 761 bp corresponded to slightly more than a quarter of a typical mastrevirus genome (Table2).

**Table 2:** Lengths, numbers of reads and BlastX analysis results for siRNA *de novo* contigs from sugarcane plant VARX with detectable homology to mastreviral sequences.

Virus	Contig	Contig length (bp)	Number of reads	BlastX Virus	BlastXLocus	BlastX e-value	Percent identity
SSEV	#121	101	270	SSEV (AAF76871)	CP	2.60E–9	100%
	#176	133	520	SSEV (AAC98080)	MP	7.22E–15	100%
SWSV	#44	117	914	SacSV (YP_003288767)	CP	7,16E–06	68%
	#79	275	7640	WDIV (YP_006273068)	MP	3,29E–11	70%
	#86	258	1649	WDIV (YP_006273069)	CP	6,78E–20	50%
	#101	111	1284	SSRV (ABZ03975)	CP	1,94E–05	64%

Acronyms used are as follows: SSEV (Sugarcane streak Egypt virus), SWSV (Sugarcane white streak virus), SacSV (Saccharum streak virus), WDIV (Wheat dwarf India virus), SSRV (Sugarcane streak Reunion virus).

Following the cloning and sequencing of the full genome of the new mastrevirus (SWSV; see below) it was determined that 0.59% of the Illumina reads obtained from the VARX plant could be mapped to this genome (Figure 1) to generate contigs that covered 96.3% of the genome at an average depth of 185X with only seven gaps of between three and 40 nucleotides. These gaps were located within the large intergenic region (three gaps) and within the probable replication associated protein (Rep) gene (four gaps; Figure 1) encoded by the C1 ORF. It is noteworthy that the ∼120 nt long region of very low coverage (<4X) identified using the VANA approach mapped to the same part of the LIR region of the SWSV genome that remained uncovered during the Illumina-based siRNA sequencing (Figure 1). As has been previously observed for other viruses, genome coverage was highly heterogeneous (Figure 1). However, a clear general trend could be observed, with the region corresponding to the virion sense V1 and V2 ORFs (encoding CP and MP proteins, respectively), showing an average coverage depth of ∼436X and the complementary sense C1 ORF showing an average coverage of only ∼38X. Coverage of the non-coding large and small intergenic regions and the presumed C1 ORF intron were even lower at 17.5X and 6.8X, respectively.

**Figure 2:**
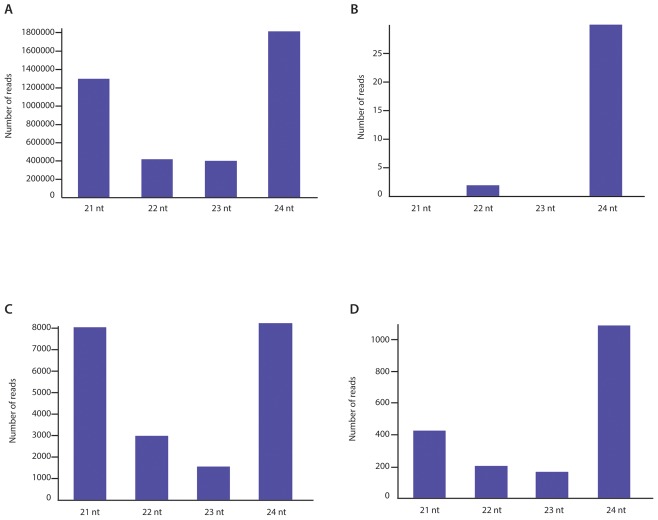
Size distribution of sequenced siRNAs obtained from the VARX plant. The histograms represent the numbers of siRNA reads in each size class. (A) The size distributions of total reads, (B) The size distributions of reads mapping to the *rep* gene C-sense intronic region of SWSV, (C) The size distributions of reads mapping to the V1–V2 ORFs region of SWSV and (D) The size distributions of reads mapping to the C1 ORF region of SWSV.

It is also noteworthy that besides differences in coverage depth, these various genomic regions of SWSV also showed differences in the siRNA size classes that they yielded. While there was an enrichment of the 21 and 22 nt siRNA size classes amongst the total siRNA reads mapping to the V1 and V2 ORFs, there was a depletion of the 21 nt siRNA size classes and an enrichment of the 24 nt size class amongst total siRNA reads mapping to the C1 ORF (Figure 2). The LIR and, to a lesser extent, the SIR showed a pattern similar to the C1 ORF region (data not shown). The C1 intron, however, had an extreme over-representation of the 24 nt size class with the other size classes being either nearly (22 nt) or totally (21 and 23 nt) absent (Figure 2).

Since the VARX plant was also infected with SSEV, a similar analysis of SSEV-derived siRNAs was performed. Mapping against the genome of SSEV (NC_001868) demonstrated that 0.17% of total reads (6572) were derived from it and that these reads covered 98.6% of the SSEV genome at an average depth of 55X, leaving only 4 gaps of between 5 and 15 nucleotides (Figure S1). Although showing some high degrees of local heterogeneity, genome coverage of SSEV was less biased when comparing the different genomic regions. Nevertheless a similar trend to that associated with SWSV was observed with a higher depth of coverage for the virion sense V1–V2 ORFs (76.5X) than for both the complementary sense C1 ORF (37.6X) and the non-coding regions (46X). Also, as for SWSV, the 21–22 nt siRNA size classes were enriched amongst those mapping to the virion sense ORFs and the 24 nt, siRNA size class was enriched amongst those mapping to the complementary sense C1 ORF (Figure S1). However, unlike for SWSV, no strong siRNA size-class biases were observed for the non-coding regions (data not shown).

By collectively using the Illumina siRNA reads and the 454 VANA reads it was possible to assemble a single genome of the novel mastrevirus from both the VARX and USDA plants.

SWSV

### Associations between siRNAs and SWSV/SSEV genomic and transcript secondary structures

It has been previously determined that nucleic acid structures can have an appreciable impact on both the distribution of siRNA targets [36], [37], and the operational efficiency of small RNA mediated anti-viral and anti-viroid defences [37], [38]. We detected strong evidence for the presence of ssDNA secondary structures in both the SWSV (30 high confidence structure set (HCSS) identified) and SSEV (29 HCSS structures identified) genomes (Table 3). The distributions of the HCSS structural elements were, however, different in the predicted virion and complementary strand transcripts of the two viruses, with only two HCCS structures detected in the SWSV complementary strand transcript and none being detected in the SSEV virion strand transcript (so that this particular transcript was not analysed further).

**Table 3:** Associations between siRNAs and SWSV/SSEV genomic and transcript secondary structures in the HCSS.

Sequence name	Component	Length	Number of structures	siRNA type	Probability if association between siRNAs and secondary structure (KS Test)	Probability of no association between siRNAs and base-paired nucleotides (WRS test)	Probability of no association between siRNAs and unpaired nucleotides (WRS test)
SWSV	Full genome	2830	30	All	**5.69×10^−5^**	0.999	**6.20×10^−4^**
				21	0.205	0.590	0.410
				22	**3.35×10^−6^**	0.992	**0.008**
				23	**0.0005**	0.999	**6.81×10^−4^**
				24	**3.48×10^−9^**	0.999	**2.67×10^−6^**
	V-strand transcript	1222	16	All	**0**	**1.80×10^−15^**	**1**
				21	**0.022**	**4.93×10^−17^**	**1**
				22	**0**	**4.1×10^−17^**	**1**
				23	**8.23×10^−14^**	**1.10×10^−16^**	**1**
				24	**0**	**6.07×10^−13^**	**1**
	C-strand transcript	1446	2	All	**2.00×10^−4^**	**0.100**	0.899
				21	**1.78×10^−7^**	**2.49×10^−6^**	0.999
				22	0.409	0.122	0.877
				23	**0.008**	**0.925**	0.075
				24	**0.003**	**0.970**	**0.029**
SSEV	Full genome	2706	29	All	**0.007**	**0.889**	0.111
				21	**0.016**	**0.737**	0.263
				22	**0.0001**	**0.999**	**0.001**
				23	0.140	0.861	0.139
				24	0.209	0.499	0.501
	V-strand transcript	1131	0	NA	NA	NA	NA
	C-strand transcript	1406	13	All	**0.002**	**0.019**	0.981
				21	0.670	0.465	0.535
				22	**0.006**	**0.025**	0.975
				23	**0.002**	**0.001**	0.999
				24	**0.041**	**0.023**	0.977

We detected a strong association between the absence of predicted secondary structures within the ssDNA SWSV genome and increased frequencies of corresponding 22, 23 and 24 nt long siRNAs (p-values <0.008; Table 3). Curiously, we found a different association when considering the predicted SWSV RNA transcripts with 21 nt siRNA reads displaying a strong tendency to correspond with nucleotide sites that were predicted to be base paired in both the virion and complementary strand transcripts (p-values <2.49×10-6) and the 22, 23 and 24 siRNA size classes displaying a similar tendency with respect to the virion strand transcript (p-values <6.07×10-13).

Similar to SWSV, for the SSEV full genome there was an association between the absence of ssDNA structural elements and increased frequencies of 22 nt siRNAs. Also similar to SWSV the 22, 23 and 24 nt long siRNAs display a significant tendency to correspond with transcript nucleotides that are base-paired within secondary structural elements.

### A novel sugarcane-infecting mastrevirus originating from the Nile region

**Figure 3:**
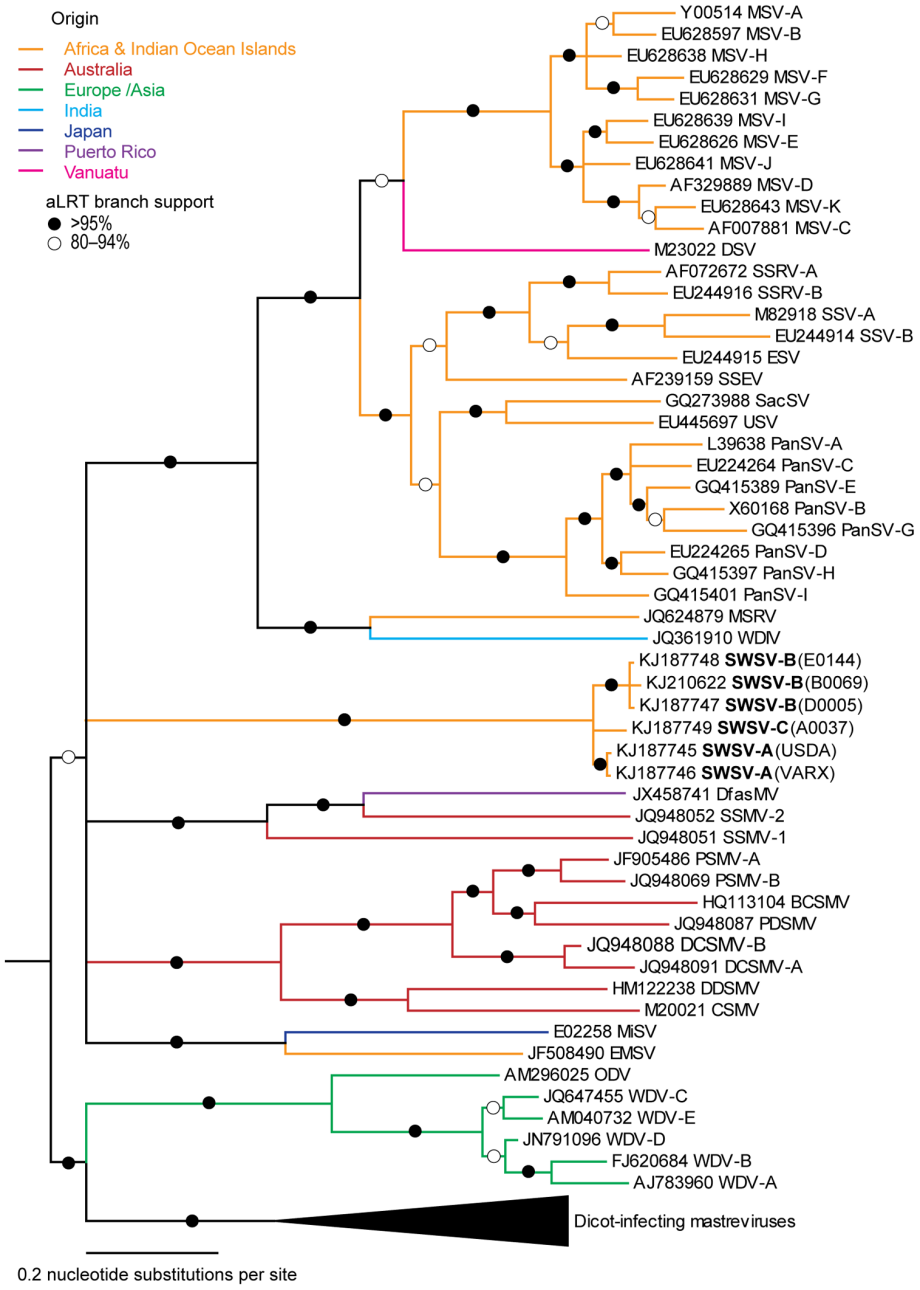
Maximum-likelihood phylogenetic tree of 63 virus isolates representing each known mastrevirus species (including major strains) and the 6 SWSV isolates determined in this study. Tree branches are coloured according to the geographical origins of the viruses. Branches marked with filled and open circles respectively have >95% and 80–94% approximate likelihood ratio test support; branches having <80% support were collapsed. The phylogenetic tree is rooted using the full genome sequence of Dicot-infecting mastreviruses.

**Figure 4:**
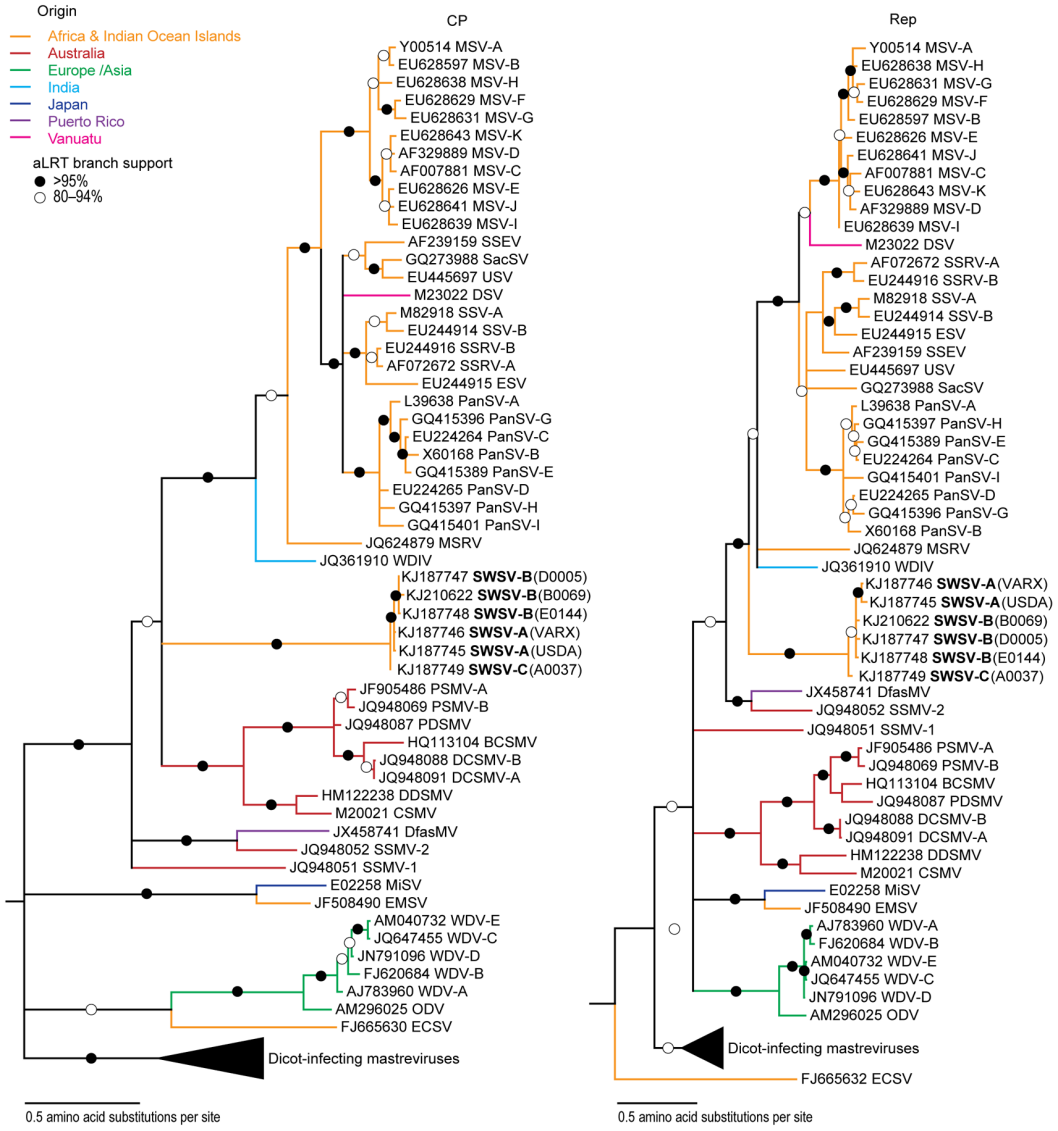
Maximum-likelihood phylogenetic tree of Rep (A) and CP (B) proteins. Tree branches are coloured according to the geographical origins of the viruses. Branches marked with filled and open circles are respectively have >95% and 80–94% approximate likelihood ratio test support; branches having <80% support were collapsed.

The complete genome of SWSV, as recovered from the VARX and USDA plants, is most similar to that of *Wheat dwarf India virus* (WDIV, Accession number NC_017828), with which it shares 61% genome-wide identity. Whereas the Rep and MP amino acid sequences of SWSV are also most similar to those of WDIV (54.4% and 44.8% identity, respectively), the CP is most similar to that of *Panicum streak virus* (PanSV, NC_001647, 51.4–53.9%). Based on the 78% species demarcation threshold set by the Geminivirus study group of the ICTV [32], it is clear that the novel mastrevirus should be considered a new species within the genus *Mastrevirus* of the Family *Geminiviridae* (Figure S2). This is further confirmed by phylogenetic analyses performed on both the full genome (Figure 3) and on the amino acid sequences of its encoded proteins (Figure 4). The new virus clearly clusters with mastreviruses on a branch that is not closely associated with any other species classified within this genus. Whereas the CP of SWSV clusters within the virus clade including the various African streak viruses, Australasian striate mosaic viruses, *Digitaria streak virus* (DSV) and WDIV, the Reps cluster with the African streak viruses and WDIV (Figure 4).

SWSV was not detected in sugarcane seedlings derived from sugarcane true seeds under sterile insect-proof conditions, in agreement with the fact that seed transmission of geminiviruses has not so far been reported. The novel mastrevirus was, however, detected in five sugarcane plants originating from Sudan (A0037, B0065, B0069, D0005 and E0144) out of the 23 screened (Table S1).

Complete SWSV genomes from four sugarcane plants (A0037, B0069, D0005 and E0144) were cloned and sequenced. The genomes of these isolates have >91% genome-wide identity with those recovered from the VARX and USDA sugarcane plants. Phylogenetic analyses of the full genomes (Figure 3) and of the amino acid sequences that they likely encode (Figure 4) confirmed that the isolates obtained from the Sudanese sugarcane plants also belong to the SWSV species. The six isolates can be further classified into 3 strains, SWSV-A (VARX, USDA), -B (B0069, D0005, E0144) and -C (A0037) (Figure S3) based on the proposed classification of mastrevirus strains outlined by Muhire et al. [32].

Using primers that allow the amplification of all sugarcane-infecting mastreviruses, including Sugarcane streak Egypt Virus, Sugarcane streak virus, Maize streak virus, Sugarcane streak Reunion virus, Eragrostis streak virus and Saccharum streak virus, the five sugarcane plants originating from Sudan were shown to be free of co-infection with other known mastreviruses. Three of them are still maintained at the CIRAD sugarcane quarantine station (B0065, B0069 and D0005) and exhibit white spots on the base of their leaf blades, around the blade joint where the two wedge shaped areas called “dewlaps” are located (Figure S4). These spots can become fused laterally, so as to appear as chlorotic stripes (Figure S4). It is noteworthy that the SWSV infected D0005 plant displayed very little evidence of these spots (only one leaf out of eight exhibited tiny white spots that resembled thrip damage) and it is therefore very likely that SWSV infections could escape visual inspection (Figure S4). Given that three of the infected sugarcane varieties exhibited mild foliar symptoms, i.e. white spots on the base of their leaf blades that become fused laterally, so as to appear as chlorotic stripes, we propose naming the new species Sugarcane white streak Virus.

### Genome analysis of SWSV

The SWSV genomes recovered from the various sugarcane plants were between 2828 and 2836 nt and are, in almost all respects, very similar to those of all other previously described mastreviruses. The one exceptional feature of the SWSV genomes is that in case of the VARX and USDA isolates alternative splicing of complementary sense transcripts likely results in the expression of both a standard Rep (which is predicted to be 396 amino acids long), and a rather unusual RepA of 418 amino acids long. This is the only known occurrence in any geminivirus of a RepA that is larger than Rep.

Given the uniqueness of this apparent genome organisation in the USDA and VARX isolates the correct identification of the intron within the complementary sense transcript was verified. RT-PCR reactions targeting the complementary sense transcript clearly indicated the presence of a mixture of spliced and non-spliced complementary sense mRNA transcripts, and confirmed that the correct locations of the acceptor and donor sites of the 66 nt long SWSV intron had been identified (Figure S5).

### Analysis of recombination

All the SWSV genome sequences determined here share evidence of the same ancestral recombination event in the short intergenic region - corresponding to genomic coordinates 1419–1468 in the USDA isolate (p = 3.821×10-7 for the GENECONV, MAXCHI and RDP methods implemented in RDP4.24). Corresponding coordinates are known to be very common sites of recombination in mastreviruses [39] and the fragment that they delimit in SWSV has apparently been derived from something resembling an African streak virus.

## Discussion

We have performed NGS-based analyses of both siRNA and VANA isolated from sugarcane plants originating from Egypt. Both sequence-independent NGS approaches revealed the presence of a novel mastrevirus, SWSV, which had so far escaped routine quarantine detection assays, possibly because it was present in mixed infection with SSEV. The procedures used for the discovery of SWSV pave the way towards the application of NGS-based quarantine detection procedures. Such procedures would likely be hierarchical with a first stage sequence-independent NGS step followed by sequence-dependent secondary assays. Whereas the first step would be to identify novel viruses within a single plant (perhaps one displaying apparent disease symptoms), the second step would be to use sequence dependent approaches to both confirm the presence of any novel virus(es) identified in the original host, and identify the presence of this(ese) virus(es) in larger plant collections. A major strength of such an approach is that it would also yield complete genome sequences.

The present study also confirms that both VANA [13] and siRNA [16] can be successfully targeted by metagenomics approaches for the discovery and characterization of plant-infecting DNA viruses. The VANA-based 454 pyrosequencing approach has several advantages as it initially combines reverse-transcriptase priming and a Klenow Fragment step, which potentially enables the detection of both RNA and DNA viruses. Additionally, up to 96-tagged amplified DNAs (cDNA and DNAs amplified using the Klenow Fragment step) can be pooled and sequenced in multiplex format [15] making this approach very useful for routine diagnostic screening of plants within BRCs and quarantine stations. However, validation using plants infected or co-infected with RNA and DNA viruses needs to be carried out in order to determine the sensitivity and specificity levels of this 454-based VANA sequencing approach.

Virus-derived siRNAs naturally accumulate in virus-infected plants as a consequence of the action of Dicer enzymes as part of the RNA silencing-based plant antiviral defences [40]. Adopting a metagenomic approach and randomly sequencing these siRNAs is therefore an extremely powerful way to discover and characterise previously unknown plant viruses and viroids [16], [41]. In addition to providing evidence for the presence of the two mastreviruses co-infecting the VARX sugarcane plant, this approach provided information on the interaction of the plant antiviral silencing machinery and these two viruses. Although these aspects have been studied previously in geminiviruses in the *Begomovirus* genus (Blevins et al., 2006; Akbergenov et al., 2006; Rodríguez-Negrete et al., 2009; Yang et al., 2011; Aregger et al., 2012), very little comparable information has previously been available for mastreviruses.

The siRNA distributions observed here for SWSV and SSEV, perhaps unsurprisingly, seem to largely parallel those previously reported for begomoviruses. In particular, the differences in size classes observed between different genome regions suggest that mastreviruses are subject both to transcriptional gene silencing, based on 24 nt long siRNAs produced through the action of DCL3, and to post-transcriptional gene silencing (PTGS) mediated by the 21–22 nt long siRNAs produced through the action of the antiviral Dicers DCL4 and DCL2 (Rodríguez-Negrete et al., 2009; Aregger et al., 2012). The action of the former mechanism is particularly evident in the siRNAs mapping to the SWSV intron but is also, to a lesser extent, evident in the siRNAs mapping to both the non-coding regions and the complementary sense ORFs of SWSV and SSEV. On the other hand, the 21–22 nt siRNA size classes associated with PTGS are particularly evident in the siRNAs mapping to the two virion sense ORFs which are known to be more actively transcribed in mastreviruses than their complementary sense counterparts [42].

For both SWSV and SSEV we detected a significant association between the frequencies of siRNAs and the presence/absence of predicted secondary structures within both the single stranded DNA (ssDNA) genomes of these viruses and their predicted single stranded RNA (ssRNA) complementary and virion strand transcripts. However, whereas significantly more siRNAs corresponded with unstructured regions of the ssDNA genome, for the transcripts significantly more siRNAs corresponded with structured regions of ssRNA. It is plausible that base-paired nucleotides within transcript RNA molecules are protected from siRNA binding and that the secondary structures evident both in transcripts produced by SWSV, SSEV and in mastrevirus genomes in general [43] may represent an evolutionary adaptation for viral persistence. In mammalian RNA viruses there is an association between degrees of genomic secondary structure and infection duration with viruses having highly structured genomes tending to cause chronic infections and viruses with unstructured genomes tending to cause acute infections [44], [45].

In both analysed Egyptian sugarcane accessions, VARX and USDA, SWSV was found to be present in co-infections with SSEV. Both sugarcane plants were independently collected in Egypt which suggests that SWSV infection of Egyptian sugarcane plants may not be a rare phenomenon. SWSV was also detected in SSEV-free plants that originated from Sudan. It is noteworthy that one of the Sudanese plants from which SWSV was isolated, E0144, was initially grown in Sudan in 1992 before being transferred to Barbados in 1998 and subsequently sent back to the CIRAD Sugarcane Quarantine Station in 2009 (unpublished data, CIRAD Sugarcane Quarantine Station). Assuming that SWSV did not infect this plant in Barbados between 1998 and 2009, it is plausible that SWSV was present along the Nile basin at least from the late 1980s. Interestingly, as a consequence of indel polymorphisms in the 66 nt long SWSV intron, the Egyptian SWSV isolates VARX and USDA have a highly unusual genome organization and likely express a RepA protein that, while having the same N- and C-terminus sequences as Rep, is 22 amino acids longer than Rep.

The recent discoveries of SWSV and other highly divergent mastreviruses [46], [47] suggest that this geminivirus genus likely encompasses a far greater diversity and has a greater global distribution than has been previously appreciated. The SWSV isolate from the Sudanese sugarcane plant that had been propagated in Barbados represents only the third instance of discovery of mastreviruses in the New World [16], [48], and suggests that there may have been other undetected recent introductions of mastreviruses to the Americas. Although insect transmission of mastreviruses in the New World remains to be reported, it is noteworthy that one of the three mastrevirus species that has so far been detected in the Americas was isolated from a dragonfly which had possibly eaten a plant feeding insect that was carrying the virus [48]. The presence of SWSV in Barbados offers an opportunity to investigate possible natural transmission of the virus by screening sugarcane planted near the SWSV infected E0144 accessions. Phylogenetic analyses of any SWSV genomes sampled from such plants should reveal their likely recent transmission histories.

Given the relatively high degrees of sequence divergence observed between the different SWSV isolates described here (∼9%), it is plausible that the natural geographical range of SWSV is broader than just the Nile basin. Also, the global dissemination of sugarcane cuttings, the absence of SWSV diagnostic tools, and the fact that SWSV induces, at least in one case, extremely mild symptoms in sugarcane imply that SWSV may have already been unknowingly distributed throughout the sugarcane growing regions of the world. The failure of established sugarcane quarantine diagnostics in this regard provides a dramatic example of how potentially pathogenic viruses can evade the screening procedures of quarantine facilities and may spread worldwide through international plant material exchanges. In this regard the situation with SWSV might closely match that of *Sugarcane yellow leaf virus* (SCYLV), which remained unnoticed for at least 30 years during its spread throughout the world [49]. In order to accurately determine the potential economic impacts of the dissemination of SWSV, additional studies assessing the pathogenicity of this virus are certainly warranted.

Our study stresses both the potential advantages of NGS-based virus metagenomic screening in a plant quarantine setting, and the need to better assess viral diversity within plants that are destined for exotic habitats. It indicates that a combination of sequence-independent NGS-based partial viral genome sequencing coupled with sequence-dependent Sanger-based full genome cloning and sequencing is likely to reduce the number of non-intercepted virus pathogens passing through plant quarantine stations, while at the same time alerting authorities to the presence and potential spread of viruses with unknown pathogenic potentials.

## Supporting Information

Figure S1 (A) Genome coverages obtained after *a posteriori* mapping against the complete genome of SSEV of reads produced by Illumina (siRNA sequencing). The genomic organization of SSEV is schematically shown at the top of the figure. (B) Size distribution of sequenced siRNAs obtained from the VARX plant mapping on the V1–V2 ORFs region of SSEV. Histograms represent the number of siRNA reads in each size class. and (C) Size distribution reads mapping on C1–C2 ORFs region of SSEV.
(TIF)

Figure S2 **Two-dimensional genome-wide percentage pairwise nucleotide identity plot of monocot-infecting mastreviruses including the six novel SWSV isolates from this study.**
(TIF)

Figure S3 (A) Maximum-likelihood phylogenetic tree of six SWSV isolates. The six isolates can be classified into 3 strains, SWSV-A (VARX, USDA), -B (B0069, D0005, E0144) and -C (A0037). (B) Genome-wide pairwise nucleotide similarity score matrix, the 94% strain demarcation threshold set by the Geminivirus study group of the ICTV (Muhire et al. 2013) is indicated (green coloured below 94% and pink-red coloured above 94%).
(TIF)

Figure S4 **Symptoms caused by SWSV on B0065, B0069 and D0005 plants.**
(TIF)

Figure S5 **Reverse transcriptase priming and amplification of nucleic acids were carried out in order to detect the** ***rep*** **gene C-sense intronic region.** (A) Agarose gel detection of presence of a mixture of spliced and non-spliced complementary sense mRNA transcripts. 1: 1 Kb ladder; 2: Reverse transcriptase priming and amplification of nucleic acids without DNase treatment of extracted RNAs; 3: Reverse transcriptase priming and amplification of nucleic acids with DNase treatment of extracted RNAs. (B) 66 nt long SWSV intron nucleotidic sequence and splice donor and acceptor sites. The sequence of the intron (in lower case) and its flanking exons (upper case) are shown. The 5′ (donor) and 3′ (acceptor) splice sites are underlined (lower case).
(TIF)

Table S1 **List of the sugarcane varieties from the CIRAD Sugarcane Quarantine Station (SQS) that were screened for the presence of all known sugarcane-infecting mastreviruses and SWSV.**
(DOC)
