# Supplementary figures and images for: Appearances Can Be Deceptive: Revealing a Hidden Viral Infection with Deep Sequencing in a Plant Quarantine Context
Source: PLoS One. 2014 Jul 25;9(7):e102945. doi: 10.1371/journal.pone.0102945 (PMC4111361; doi:10.1371/journal.pone.0102945)

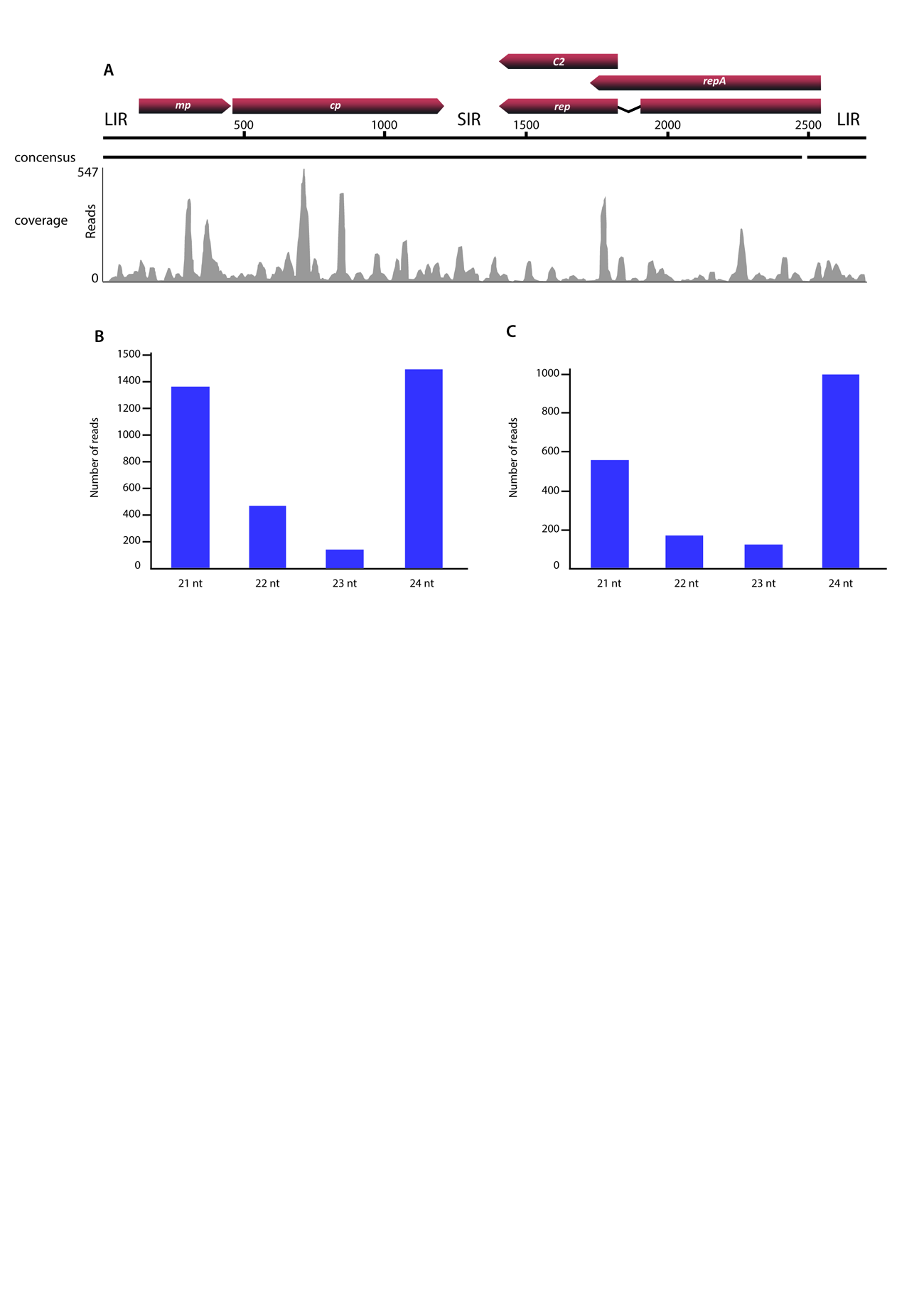

Supplement: Figure S1 — (A) Genome coverages obtained after a posteriori mapping against the complete genome of SSEV of reads produced by Illumina (siRNA sequencing). The genomic organization of SSEV is schematically shown at the top of the figure. (B) Size distribution of sequenced siRNAs obtained from the VARX plant mapping on the V1–V2 ORFs region of SSEV. Histograms represent the number of siRNA reads in each size class. and (C) Size distribution reads mapping on C1–C2 ORFs region of SSEV. (TIF) [file pone.0102945.s001.tif]

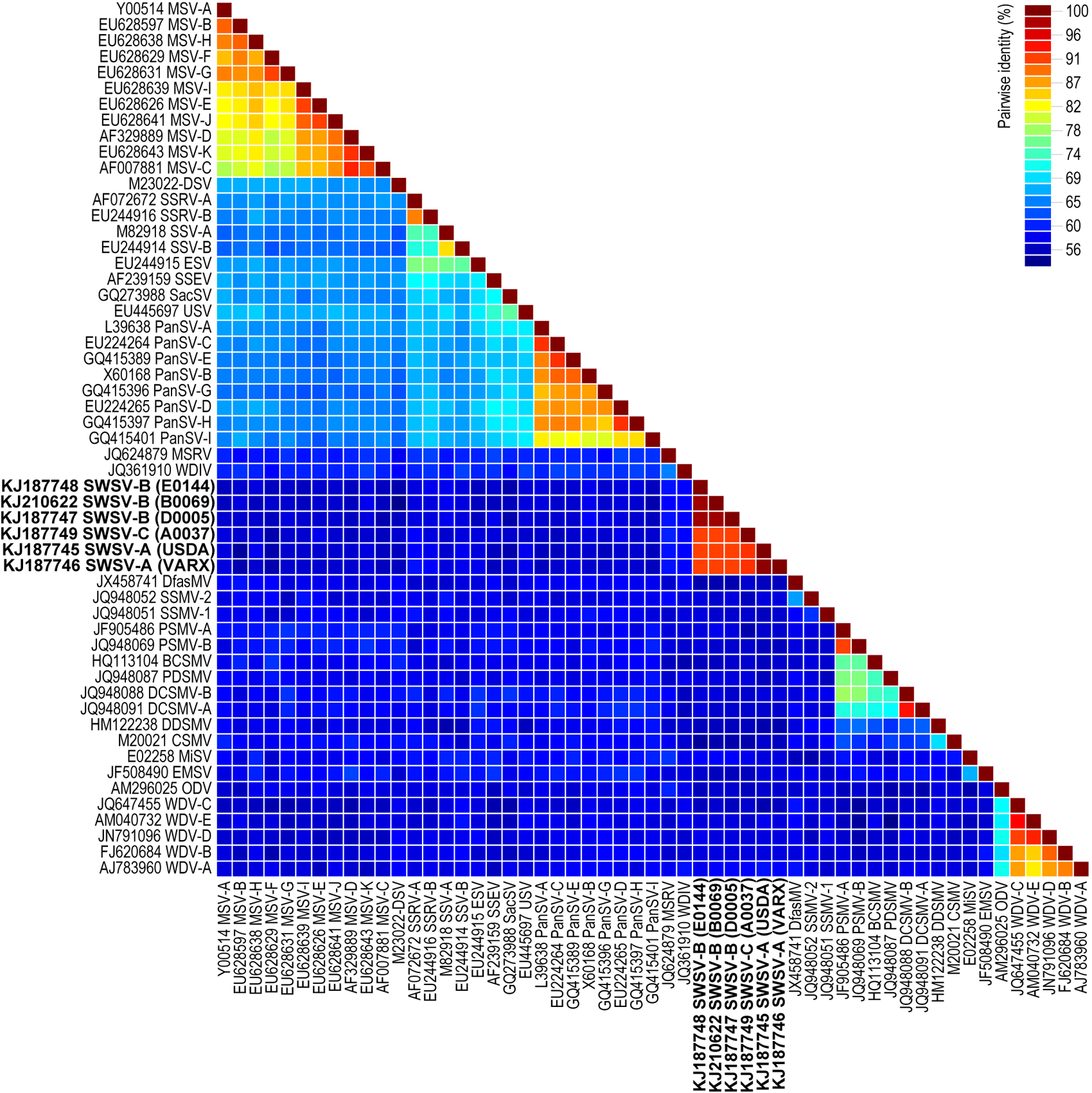

Supplement: Figure S2 — Two-dimensional genome-wide percentage pairwise nucleotide identity plot of monocot-infecting mastreviruses including the six novel SWSV isolates from this study. (TIF) [file pone.0102945.s002.tif]

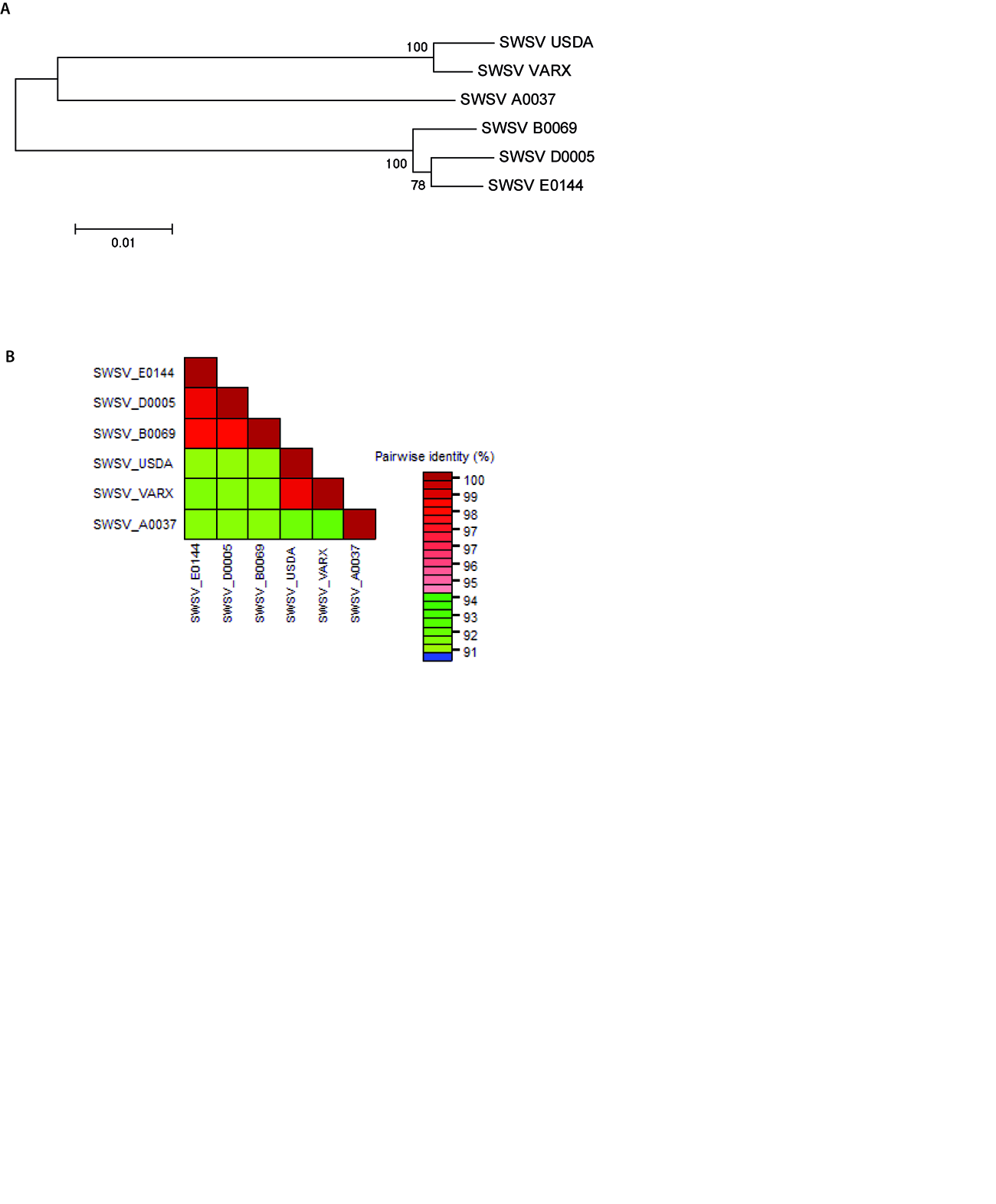

Supplement: Figure S3 — (A) Maximum-likelihood phylogenetic tree of six SWSV isolates. The six isolates can be classified into 3 strains, SWSV-A (VARX, USDA), -B (B0069, D0005, E0144) and -C (A0037). (B) Genome-wide pairwise nucleotide similarity score matrix, the 94% strain demarcation threshold set by the Geminivirus study group of the ICTV (Muhire et al. 2013) is indicated (green coloured below 94% and pink-red coloured above 94%). (TIF) [file pone.0102945.s003.tif]

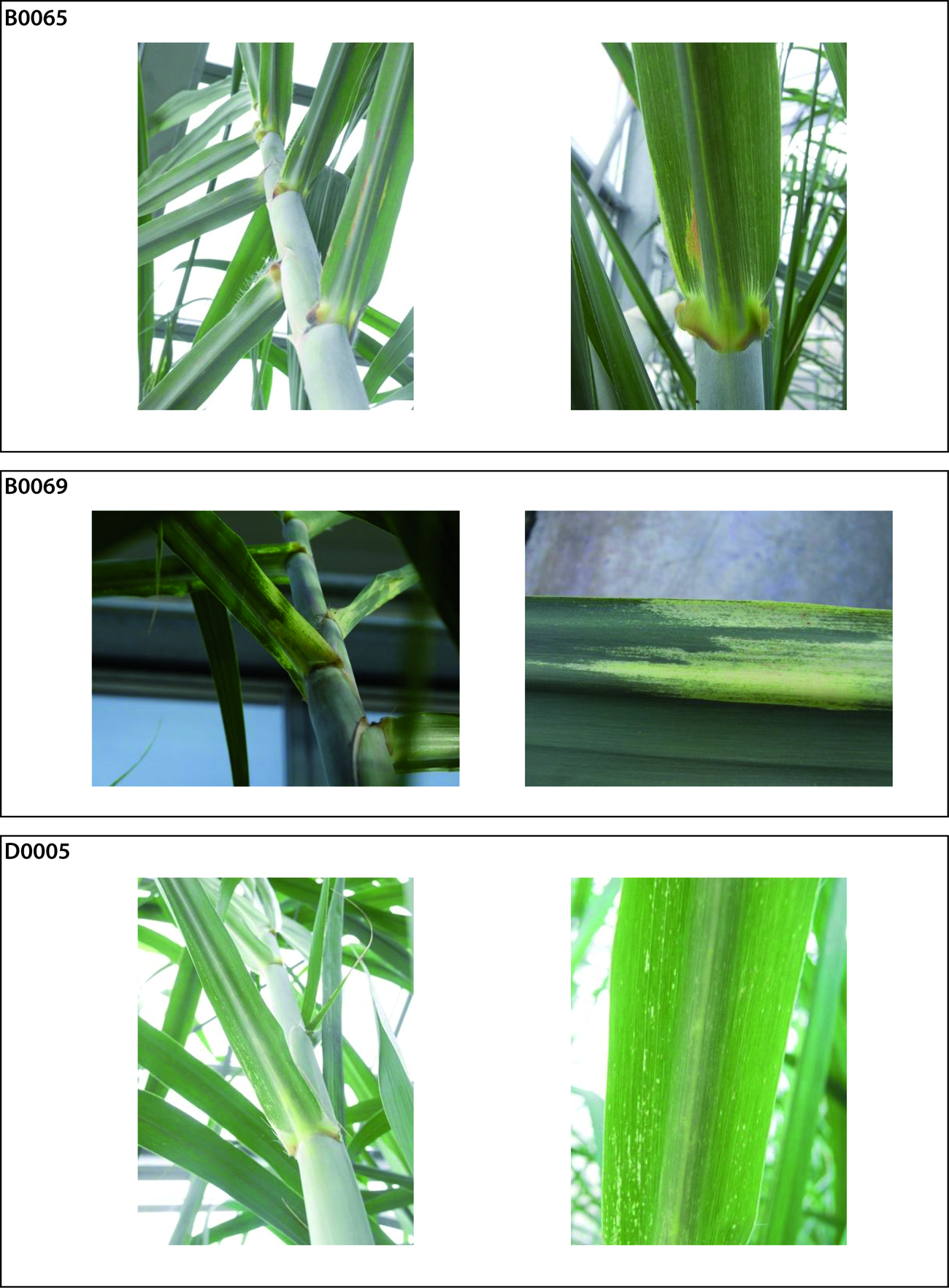

Supplement: Figure S4 — Symptoms caused by SWSV on B0065, B0069 and D0005 plants. (TIF) [file pone.0102945.s004.tif]

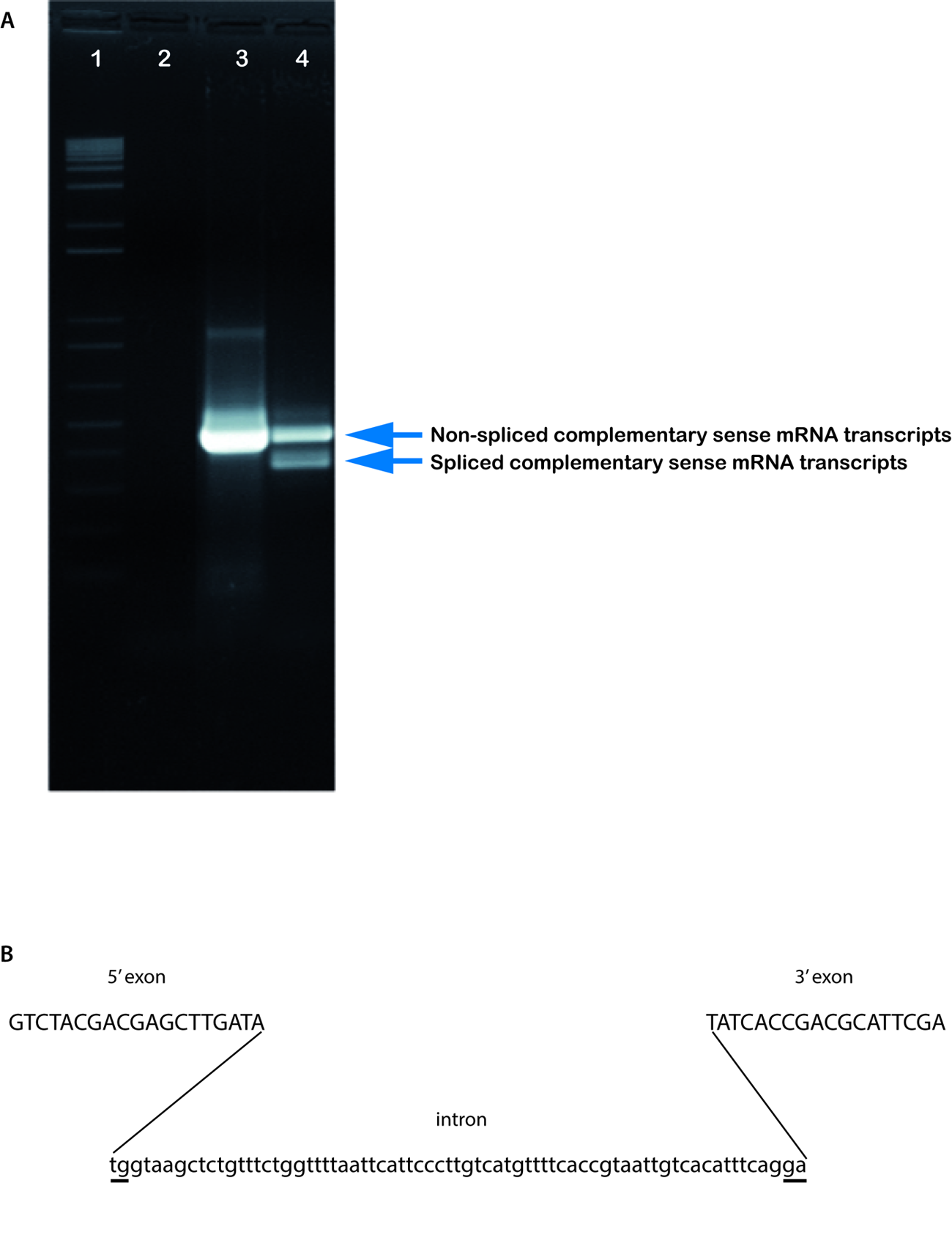

Supplement: Figure S5 — Reverse transcriptase priming and amplification of nucleic acids were carried out in order to detect the rep gene C-sense intronic region. (A) Agarose gel detection of presence of a mixture of spliced and non-spliced complementary sense mRNA transcripts. 1: 1 Kb ladder; 2: Reverse transcriptase priming and amplification of nucleic acids without DNase treatment of extracted RNAs; 3: Reverse transcriptase priming and amplification of nucleic acids with DNase treatment of extracted RNAs. (B) 66 nt long SWSV intron nucleotidic sequence and splice donor and acceptor sites. The sequence of the intron (in lower case) and its flanking exons (upper case) are shown. The 5′ (donor) and 3′ (acceptor) splice sites are underlined (lower case). (TIF) [file pone.0102945.s005.tif]
