# Supplementary material for: Appearances Can Be Deceptive: Revealing a Hidden Viral Infection with Deep Sequencing in a Plant Quarantine Context
Source: PLoS One. 2014 Jul 25;9(7):e102945. doi: 10.1371/journal.pone.0102945 (PMC4111361; doi:10.1371/journal.pone.0102945)
Supplement: Table S1 — List of the sugarcane varieties from the CIRAD Sugarcane Quarantine Station (SQS) that were screened for the presence of all known sugarcane-infecting mastreviruses and SWSV. (DOC) [file pone.0102945.s006.doc]

**Table S1: List of the sugarcane varieties from the CIRAD Sugarcane Quarantine Station (SQS) that were screened for the presence of all known sugarcane-infecting mastreviruses and SWSV.**

| SQS number | Other number | Origin of sugarcane | Country from which sugarcane varieties were imported to SQS | Year of entrance in SQS | Sugarcane-infecting mastrevirus |
| --- | --- | --- | --- | --- | --- |
| VAR X |  | Egypt | Egypt | 1999 | SSEV  SWSV |
| USDA |  | Egypt | USA (USDA-APHIS Plant Germplasm Quarantine) | 2007 | SSEV  SWSV |
| Soudan_3.11.04 |  | Sudan | Sudan | 2004 |  |
|  | KN 93 08 | Sudan | Sudan | 2000 |  |
| E0144 | KNB 92 101 | Sudan | Barbados | 2009 | SWSV |
| A0033 | KNB 96 64 | Sudan | Sudan | 2004 |  |
| A0034 | KNB 96 65 | Sudan | Sudan | 2004 |  |
| A0035 | KNB 96 49 | Sudan | Sudan | 2004 |  |
| A0036 | KNB 96 98 | Sudan | Sudan | 2004 |  |
| A0037 | KNB 96 108 | Sudan | Sudan | 2004 | SWSV |
| A0038 | KNB 96 146 | Sudan | Sudan | 2004 |  |
| A0039 | KNB 96 158 | Sudan | Sudan | 2004 |  |
| A0040 | KNB 96 207 | Sudan | Sudan | 2004 |  |
| A0041 | KNB 96 211 | Sudan | Sudan | 2004 |  |
| A0042 | KNB 96 223 | Sudan | Sudan | 2004 |  |
| B0065 | KN 88 104 | Sudan | Sudan | 2005 | SWSV |
| B0066 | KN 88 136 | Sudan | Sudan | 2005 |  |
| B0067 | KN 88 260 | Sudan | Sudan | 2005 |  |
| B0068 | KN 89 21 | Sudan | Sudan | 2005 |  |
| B0069 | KN 89 24 | Sudan | Sudan | 2005 | SWSV |
| D0001 | KN 87 012 | Sudan | Sudan | 2007 |  |
| D0002 | KN 87 065 | Sudan | Sudan | 2007 |  |
| D0003 | KN 88 015 | Sudan | Sudan | 2007 |  |
| D0004 | KN 88 043 | Sudan | Sudan | 2007 |  |
| D0005 | KN 88 147 | Sudan | Sudan | 2007 | SWSV |
| X01081 |  | Guadeloupe (French West Indies) (seeds) | Guadeloupe (French West Indies) |  |  |
